# A comprehensive interaction study provides a potential domain interaction network of human death domain superfamily proteins

**DOI:** 10.1038/s41418-021-00796-x

**Published:** 2021-05-15

**Authors:** Wei Zhou, Naoe Kaneko, Tomoya Nakagita, Hiroyuki Takeda, Junya Masumoto

**Affiliations:** 1 Department of Analytical Pathology, Ehime University Graduate School of Medicine, Toon, Ehime Japan; 2 Division of Proteo-Drug-Discovery Sciences, Ehime University Proteo-Science Center, Matsuyama, Ehime Japan; 3 Division of Pathology, Ehime University Proteo-Science Center, Toon, Ehime Japan

**Keywords:** Cell death and immune response, Rheumatic diseases

## Abstract

Human death domain superfamily proteins (DDSPs) play important roles in many signaling pathways involved in cell death and inflammation. Disruption or constitutive activation of these DDSP interactions due to inherited gene mutations is closely related to immunodeficiency and/or autoinflammatory diseases; however, responsible gene mutations have not been found in phenotypical diagnosis of these diseases. In this study, we comprehensively investigated the interactions of death-fold domains to explore the signaling network mediated by human DDSPs. We obtained 116 domains of DDSPs and conducted a domain–domain interaction assay of 13,924 reactions in duplicate using amplified luminescent proximity homogeneous assay. The data were mostly consistent with previously reported interactions. We also found new possible interactions, including an interaction between the caspase recruitment domain (CARD) of CARD10 and the tandem CARD–CARD domain of NOD2, which was confirmed by reciprocal co-immunoprecipitation. This study enables prediction of the interaction network of human DDSPs, sheds light on pathogenic mechanisms, and will facilitate identification of drug targets for treatment of immunodeficiency and autoinflammatory diseases.

## Introduction

The human death domain (DD) superfamily is one of the largest and most studied domain superfamilies. It comprises four subfamilies called the DD subfamily, the death effector domain (DED) subfamily, the caspase recruitment domain (CARD) subfamily, and the pyrin domain (PYD) subfamily [1, 2]. Death domain superfamily proteins (DDSPs) are characterized by containing death-fold domains (DFDs) and function in various signaling pathways involved in apoptosis and inflammation by assembling oligomeric complexes via homotypic binding and inducing caspase and/or kinase activation [3].

Genetic mutations in DFD-containing proteins often cause various immunodeficiency and autoinflammatory diseases [4]. For example, Fas-associated death domain (FADD) interacts with Fas through its DD and recruits pro-caspase‐8 through its DED to form the death-inducing signaling complex (DISC) [5]. Mutations of Fas that lead to the disruption of DISC formation cause autoimmune lymphoproliferative syndrome [6, 7]. NLRP3 interacts with apoptosis-associated speck-like protein containing a CARD (ASC) through its PYD and recruits pro-caspase‐1 through the CARD of ASC to form the inflammasome. Mutations of NLRP3 that lead to constitutive activation of the inflammasome cause an autoinflammatory disease called cryopyrin-associated periodic syndrome [8, 9]. Many autoinflammatory diseases are regarded as rare diseases, with few patients, and their pathogenesis has not been fully elucidated [10, 11].

Although much evidence have been accumulated, no mutations of responsible genes for these diseases have been identified, even in phenotypically diagnosed cases [12]. This prompted us to comprehensively analyze the interactions between all DDSPs, which may provide clues to decipher the pathways and factors associated with immunodeficiency and autoinflammatory diseases.

**Fig. 1:**
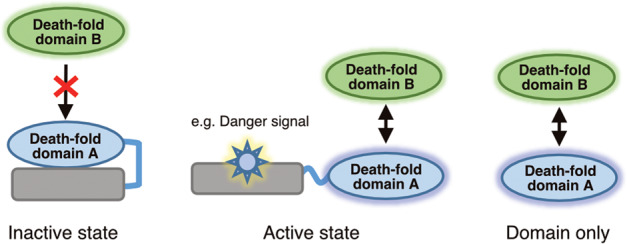
Domains are more suitable than full-length proteins for studying domain–domain interactions between DDSPs. Full-length DDSPs are inactive in the absence of upstream signals. Domains are theoretically expected to bind to their partner domains without interference.

In this study, we focused on domain–domain interactions rather than on full-length protein–protein interactions. As shown in the diagram in Fig. 1, many DDSPs are inactive in the absence of upstream signals and therefore fail to interact with their partner proteins [13]. However, the domains of DDSPs are theoretically expected to be able to bind to the domains of their partners without interference.

We synthesized FLAG- and biotin-tagged recombinant DDs, DEDs, CARDs, and PYDs using the wheat germ cell-free synthesis system and investigated domain–domain interactions using the amplified luminescence proximity homogeneous assay (ALPHA) to elucidate the interaction network of DDSPs.

## Materials and methods

### Collection of cDNA clones encoding human DDSP domains

cDNA clones encoding human DDSP domains (Supplementary Table S1) were collected from the cDNA resources of the Kazusa DNA Research Institute [14], the Mammalian Gene Collection [15], and the nonprofit repository of Addgene.

### Construction of tagged recombinant cDNA plasmids

Genes encoding DDSP domains were amplified by PCR using cDNA as templates. Overlapping sequences were added at the 5′ and 3′ ends for seamless cloning. DNA fragments encoding 27 DDSP domains were prepared by the GeneArt Gene Synthesis service (Thermo Fisher Scientific, Waltham, MA, USA). Amplified DNA fragments were subcloned into the pEU-E01-GW-FLAG and pEU-E01-GW-bls vectors using Gibson Assembly seamless cloning. After subcloning, pEU expression plasmids were arranged in a 96-well format and stored as glycerol stocks. The glycerol stocks were diluted with TE buffer and used as templates of PCR. Template DNA fragments for transcription were PCR-amplified using the SPu-2 primer (5′-CAGTAAGCCAGATGCTACAC-3′) and AODA2306 primer (5′-AGCGTCAGACCCCGTAGAAA-3′).

### Preparation of recombinant DDSP domains using a wheat germ cell-free synthesis system

The tagged recombinant human DDSP domains were synthesized using a wheat germ cell-free synthesis system [16]. Transcription and translation reactions were conducted using a WEPRO7240 Expression Kit (CellFree Sciences, Matsuyama, Japan). The transcription reaction mixture was prepared by mixing 2.5 µL of transcription buffer LM, 1.25 µL of NTP mixture (25 mM each), 0.25 µL of RNase inhibitor, 0.5 µL of SP6 polymerase, and 2.5 µL of PCR product in a 96-well plate. The transcription reaction was incubated at 37 °C for 18 h. Twenty-five microliters of the translation mixture containing 12.5 µL of mRNA, 8 µL of WEPRO 7240 wheat germ extract, 0.1 µL of creatine kinase (20 mg/mL) (Roche Diagnostics, Basel, Switzerland), and 0.5 µL of RNase inhibitor was prepared and overlaid with 125 µL of translation buffer (SUB-AMIX SGC) in a 96-well plate. The biotin ligation site was biotinylated enzymatically by adding BirA biotin ligase and biotin (Sigma-Aldrich, St. Louis, MO, USA) to the translation mixture [17]. The plate containing the translation reaction was incubated at 15 °C for 24 h.

### Enzyme-linked immunosorbent assay (ELISA)

Cell-free synthesized DDSP domains were diluted 20-fold, injected into a 96-well MaxiSorp plate (Nunc, Rochester, NY, USA), and incubated overnight at 4 °C. After washing with Tris-buffered saline containing 0.1% Tween 20 (TBST), the plate was blocked with TBST containing 5% skimmed milk for 1 h at room temperature. Next, the plate was incubated with an anti-DYKDDDDK tag monoclonal antibody (012–22384, FUJIFILM Wako Pure Chemical, Osaka, Japan) or anti-biotin antibody (A4541, Sigma-Aldrich) diluted 1:2000 and 1:1000, respectively, in TBST containing 5% skimmed milk for 1 h at room temperature. Thereafter, the plate was washed three times with TBST and incubated with a horseradish peroxidase-conjugated anti-mouse IgG secondary antibody (GE Healthcare, Chicago, IL, USA) diluted in TBST containing 5% skimmed milk for 1 h at room temperature. Finally, 50 µL of tetramethylbenzidine liquid substrate (Sigma-Aldrich) was injected into the plate and incubated for 15–30 min at room temperature. The reaction was terminated by injecting the same volume of 1 M HCl. Absorbance at 450 nm was measured using a SpectraMAX M3 plate reader (Molecular Devices, San Jose, CA, USA).

### Amplified luminescence proximity homogeneous assay

All ALPHA reactions were conducted in an AlphaPlate-384 microplate (PerkinElmer, Waltham, MA, USA). All proteins and reagents were diluted in reaction buffer [100 mM Tris-HCl (pH 8.0), 0.01% Tween 20, and 1 mg/mL bovine serum albumin]. Twenty microliters of solution containing 0.4 μL of a biotin-tagged domain in reaction buffer was dispensed into the reaction plate (two domains per plate, 192 replicates) using a Viaflo automated multichannel pipette and the Viaflo Assist system (Integra, Hudson, NH, USA). Next, 0.4 µL of FLAG-tagged domain was transferred to the reaction plate (96 domains per plate, four replicates) using the Janus automated dispensing workstation (PerkinElmer) and Nanohead, a 384-well micro-syringe head (PerkinElmer). This procedure allowed 192 combinations of FLAG- and biotin-tagged domains to be mixed together in duplicate per assay plate. Then, 9.6 μL of detection mixture containing 0.02 μL of an anti-DYKDDDDK tag monoclonal antibody, 0.06 μL of streptavidin-conjugated AlphaScreen donor beads, and 0.06 μL of protein A-conjugated AlphaScreen acceptor beads in reaction buffer was added to each well of the reaction plate using a FlexDrop dropper (PerkinElmer). The detailed dispensing scheme and well layout are shown in Supplementary Fig. S1. After incubation at 25 °C for 24 h, the ALPHA chemiluminescence signal was detected by an EnVision Multilabel Plate Reader (PerkinElmer). The signal data obtained were exported to Microsoft Excel, and the median values of duplicate reactions were calculated. For the heat map, the color scale feature of Microsoft Excel was used to visualize the signal strength. MA plots and bubble charts were drawn using DataGraph (http://www.visualdatatools.com/DataGraph/).

### Immunoprecipitation

Genes encoding DDSP domains were inserted into the pcDNA3 mammalian expression vector with a FLAG or a 3×AGIA tag [18] at the C-terminus. HEK293T cells were maintained in Dulbecco’s Modified Eagle’s Medium (Thermo Fisher Scientific) supplemented with 10% heat-inactivated fetal bovine serum, penicillin, and streptomycin. Transfection was performed using the calcium phosphate method. Briefly, plasmids were diluted in 440 μL of distilled water and 60 μL of 2 M CaCl_2_, mixed with 500 μL of 2× HEPES buffer [50 mM HEPES (pH 7.00), 280 mM NaCl, and 1.5 mM Na_2_HPO_4_], and added to each 10 mL well containing HEK293T cells. A total of 1 × 10^6^ HEK293T cells were transfected with 1 μg of each of the following expression plasmid sets: pcDNA3-AIM2-PYD-AGIA and pcDNA3-NLRP9-PYD-FLAG, pcDNA3-AIM2-PYD-AGIA and pcDNA3-ASC-CARD-FLAG, pcDNA3-NLRC4-CARD-AGIA and pcDNA3-ASC-CARD-FLAG, and pcDNA3-NOD2-CARD1-CARD2-AGIA and pcDNA3-CARD10-CARD-FLAG. Transfected cells were lysed in 1000 μL of NP-40 buffer [1% Nonidet P-40, 142.5 mmol/L KCl, 5 mmol/L MgCl_2_, 10 mmol/L HEPES (pH 7.6), and 1 mmol/L ethylenediaminetetraacetic acid] supplemented with a cOmplete Mini Protease Inhibitor Cocktail tablet (Roche Diagnostics). Cell lysates were centrifuged. Supernatants were mixed with an anti-AGIA tag monoclonal antibody (in-house made) [18] or with an anti-FLAG M2 monoclonal antibody (F3165, Sigma-Aldrich) together with protein A beads (Invitrogen) and incubated for 3 h at 4 °C. The beads were washed with NP-40 buffer and precipitates were subjected to SDS-PAGE and immunoblotting. Blotting membranes were incubated with the anti-FLAG M2 monoclonal antibody or an anti-AGIA tag monoclonal antibody.

## Results

### Human DDSP domain expression plasmids were constructed

Tagged expression plasmids were constructed for cell-free synthesis of DDSP domains. We first collected the cDNA sequences of human DFD-containing proteins from public databases such as RefSeq (https://www.ncbi.nlm.nih.gov/refseq/) and UniProt (https://www.uniprot.org/). Next, we extracted the DFD fragments using their annotations and domain prediction tools including PROSITE (https://prosite.expasy.org/) and SMART (http://smart.embl-heidelberg.de/). Eventually, we identified 108 DDSP domains, including 36 DDs, 11 DEDs, 39 CARDs, and 22 PYDs. In addition, there were eight DDSP domains containing tandem DFDs, such as CARD–CARD, DED–DED, CARD–DD, PYD–CARD, and DED–DD. Including all such tandem domains, the total number of DDSP domains in this study was 116 (Supplementary Table S1). We constructed cell-free expression plasmids to synthesize all the domains tagged with FLAG or biotin at the C-terminus.

### Human DDSP domains were synthesized using the wheat germ cell-free synthesis system

Using the wheat germ cell-free protein synthesis system and expression plasmids, we synthesized 232 recombinant DDSP domains, including 116 FLAG-tagged and 116 biotin-tagged domains. In addition, we used dihydrofolate reductase (DHFR) and Venus fluorescent protein (Venus) tagged with FLAG and biotin respectively as control proteins for cell-free synthesis and ALPHA [19, 20].

**Fig. 2:**
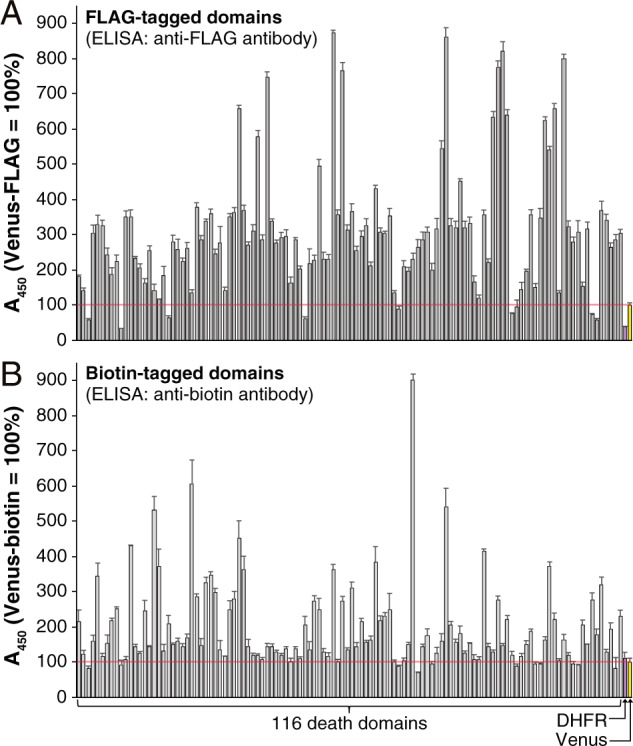
Expression levels of DDSP domains were evaluated by ELISA. DHFR and Venus were used as positive controls. The longitudinal axis shows the ELISA values representing expression levels and the horizontal axis shows the 116 domains and 2 controls listed in Supplementary Table S2. **A** Expression levels of 116 FLAG-tagged domains and 2 FLAG-tagged controls. **B** Expression levels of 116 biotin-tagged domains and 2 biotin-tagged controls.

To evaluate the expression levels of DDSP domains, ELISAs were performed using an anti-FLAG antibody (Fig. 2A) and an anti-biotin antibody (Fig. 2B), respectively (Supplementary Table S2). All the domains, both FLAG- and biotin-tagged, were expressed at a level equal to or higher than expression of DHFR, which was used as a positive control. Furthermore, the expression levels of the DDSP domains were 50–900% of that of Venus, indicating that all the DDSP domains were sufficiently expressed for the comprehensive interaction assay.

### Human DDSP domain interactions were comprehensively analyzed by ALPHA

A total of 13,924 domain–domain reactions [118 FLAG-tagged domains (116 DDSP domains plus 2 negative controls) × 118 biotin-tagged domains (116 DDSP domains plus 2 negative controls)] were conducted in duplicate in the ALPHA assay.

**Fig. 3:**
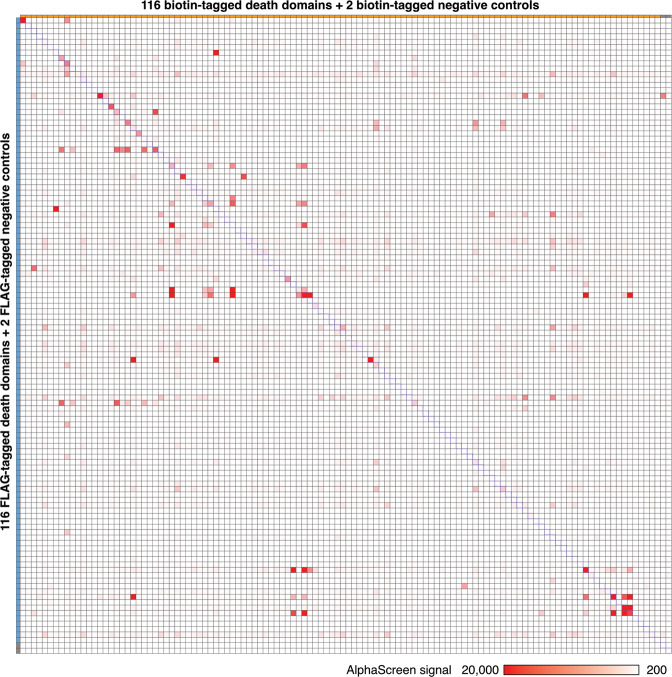
Heat map displays all the ALPHA results. There were 13,924 reactions between FLAG-tagged (116 domains plus 2 negative controls) and biotin-tagged (116 domains plus 2 negative controls) domains. The longitudinal axis shows FLAG-tagged domains and the horizontal axis shows biotin-tagged domains. The position of each point indicates the specific domain pair. Red points represent positive domain–domain interactions. The shade of red indicates the median value of two repeats in the ALPHA.

The results are listed in Supplementary Table S3, which shows the median ALPHA signals detected in two repeats. The highest value was 119,174 relative luminescence units (RLU) (APAF1_CARD-FLAG × CASP9_CARD-biotin) and the lowest value was 166 RLU. The median value was 296 RLU. A total of 799 pairs had signals higher than 1 × 10^3^ RLU, among which 236 pairs had signals higher than 2 × 10^3^ RLU and 95 pairs had signals higher than 5 × 10^3^ RLU (Fig. 3 and Supplementary Table S3).

To clarify the distribution and strength of the interactions, the values in Supplementary Table S3 were visualized as a heat map (Fig. 3). The longitudinal axis shows FLAG-tagged domains and the horizontal axis shows biotin-tagged domains. The position of each point indicates the combination of each domain–domain pair. The color of each point indicates the strength of ALPHA signals, indicating possible interactions between DDSP domains. Darker shades of red indicate stronger ALPHA signals, which are highly suggestive of interactions between the specific domains. White, which accounts for the majority of the heat map, indicates that an interaction was not detected.

**Fig. 4:**
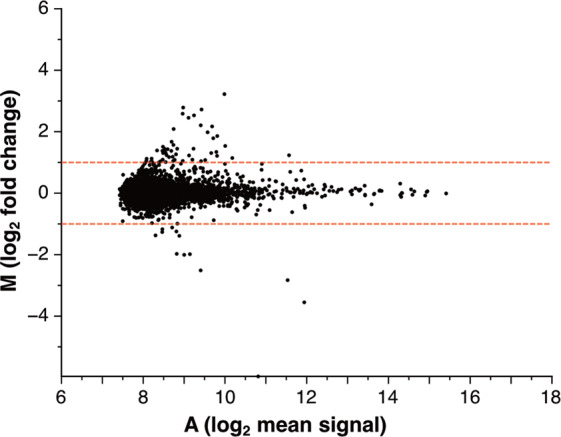
The ALPHA is highly reproducible between two repeats. An MA plot shows the reproducibility of 13,924 reactions between two repeats in the ALPHA. Each point represents the log_2_ fold change (0.5–2) in two repeats. The red dashed lines represent the fold change threshold (±1). A total of 98.74% of the points were located between the two red dashed lines, demonstrating that this assay was highly reproducible and reliable.

We assessed the reproducibility of the assay between two repeats using an MA plot (Fig. 4). Each point represents the log_2_ fold change (0.5–2) in two repeats. The red dashed lines represent the fold change threshold (±1). A total of 98.74% of the points were located between the two red dashed lines. This shows that more than 98% of the reactions were reproducible, demonstrating that this assay was highly reproducible and reliable.

### More positive interactions tend to be from the self-interacting pairs than those nonself-interacting ones

Human DDSP domains function in various signaling pathways involved in cell death and inflammation by oligomerizing with each other, which promotes interactions between proteins [2]. In this assay, therefore, we particularly compared self-interacting and nonself-interacting pairs. The 116 self-interacting pairs are located along the diagonal in Fig. 3, including six DDSPs containing homogeneous tandem DFDs (No. 028, 035, 039, 057, 092, and 102) and three DDSPs containing heterogeneous tandem DFDs (Nos. 010, 041, and 052). Among the 116 self-interacting pairs, the interactions of 38, which accounts for 32.8%, were strong with ALPHA signals higher than 1 × 10^3^ RLU. Meanwhile, among the 13,340 nonself-interacting pairs located on either side of the diagonal, the interactions of 761 (5.7%) were strong with ALPHA signals higher than 1 × 10^3^ RLU. Comparison of the percentages of positive results between self-interacting (32.8%) and nonself-interacting (5.7%) pairs implies that self-interacting pairs, which interact via homotypic binding, tend to interact more readily than nonself-interacting pairs.

### Symmetrically distributed nonself-interacting pairs in the heat map confirm previously identified heterotypic interacting pairs and indicate new interacting pairs

**Fig. 5:**
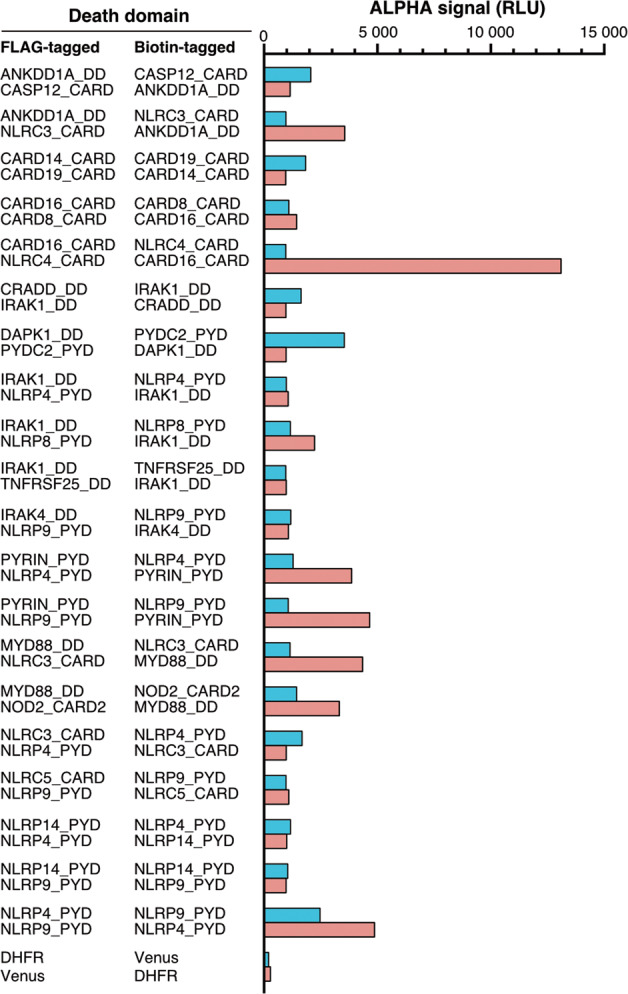
Twenty novel putative interactions were identified in this study. Double-sided interacting pairs are indicated. The bars indicate the ALPHA signals detected using various combinations of FLAG- and biotin-tagged proteins.

We focused on the red points of nonself-interacting pairs symmetrically distributed on both sides of the diagonal in the heat map (Fig. 3, hereafter called double-sided interactions). They correspond to interactions of nonself-interacting pairs that were unaffected by swapping the tag (FLAG and biotin), indicating they are highly reliable interactions. All such double-sided interactions with ALPHA signals higher than 1 × 10^3^ RLU irrespective of the tags used (FLAG × biotin and biotin × FLAG) were extracted and listed in Table 1. Among the 57 pairs, 39 were homotypic and 11 were heterotypic. Thirty-seven (65%) interactions have been previously reported, including 29 homotypic domain interactions and 1 heterotypic domain interaction, demonstrating the high reliability of the results (Table 1). The remaining ten homotypic domain interactions (CARD14_CARD × CARD19_CARD, CARD16_CARD × CARD8_CARD, CARD16_CARD × NLRC4_CARD, CRADD_DD × IRAK1_DD, IRAK1_DD × TNFRSF25_DD, PYRIN_PYD × NLRP4_PYD, PYRIN_PYD × NLRP9_PYD, NLRP14_PYD × NLRP4_PYD, NLRP14_PYD × NLRP9_PYD, and NLRP4_PYD × NLRP9_PYD) and ten heterotypic domain interactions (ANKDD1A_DD × CASP12_CARD, ANKDD1A_DD × NLRC3_CARD, DAPK1_DD × PYDC2_PYD, IRAK1_DD × NLRP4_PYD, IRAK1_DD × NLRP8_PYD, IRAK4_DD × NLRP9_PYD, MYD88_DD × NLRC3_CARD, MYD88_DD × NOD2_CARD2, NLRC3_CARD × NLRP4_PYD, and NLRC5_CARD × NLRP9_PYD) have not been previously reported (Fig. 5). These interactions must be confirmed by in vitro and in vivo studies.

**Table 1:** Double-sided interacting pairs.

Combination of death domains					ALPHA signal (RLU)			Interaction				
DFD 1		DFD 2		Homo/hetero				VaProS (updated: December 8, 2020)		BioGRID (build 4.1.190)		Others
No.	Name	No.	Name		DFD 1: DFD 2:	FLAG Biotin	Biotin FLAG	Description	PubMed ID	Interaction confirmed by	PubMed ID	Description/PubMed ID
001	AIM2_PYD	009	ASC_PYD	Homotypic		8602	4522	Association	24630722	N/A		AIM2 inflammasome
005	ANKDD1A_DD	029	CASP12_CARD	Heterotypic		2058	1148	N/A		N/A		Not found
005	ANKDD1A_DD	071	NLRC3_CARD	Heterotypic		964	3554	N/A		N/A		Not found
007	APAF1_CARD	036	CASP9_CARD	Homotypic		119,174	104,846	Direct interaction	9390557	Affinity Capture-MS	17643375	Apoptosome
008	ASC_CARD	018	CARD16_CARD	Homotypic		1972	2184	N/A		N/A		25973362
008	ASC_CARD	025	CASP1_CARD	Homotypic		1924	11,030	Direct interaction	24630722	Affinity Capture-Western	26121674	Inflammasome
008	ASC_CARD	072	NLRC4_CARD	Homotypic		2690	12,412	Association	12646168	Two-hybrid	11374873	NLRC4 inflammasome
010	ASC_PYD-CARD	025	CASP1_CARD	Homotypic		1728	4880	Direct interaction	24630722	Affinity Capture-Western	26121674	Inflammasome
010	ASC_PYD-CARD	072	NLRC4_CARD	Homotypic		1688	4502	Association	12646168	Two-hybrid	11374873	NLRC4 inflammasome
017	CARD14_CARD	021	CARD19_CARD	Homotypic		1834	956	N/A		N/A		Not found
018	CARD16_CARD	023	CARD8_CARD	Homotypic		1090	1434	N/A		N/A		Not found
018	CARD16_CARD	025	CASP1_CARD	Homotypic		14,594	11,976	N/A		Affinity Capture-MS	31091453	11432859
018	CARD16_CARD	072	NLRC4_CARD	Homotypic		958	13,094	N/A		N/A		Direct interaction not approved
019	CARD17_CARD	025	CASP1_CARD	Homotypic		1516	9228	Direct interaction	27043298	N/A		15383541
020	CARD18_CARD	025	CASP1_CARD	Homotypic		1458	12,258	Direct interaction	27043298	N/A		11051551
023	CARD8_CARD	025	CASP1_CARD	Homotypic		1610	11,702	N/A		N/A		11821383
028	CASP10_DED1-DED2	035	CASP8_DED1-DED2	Homotypic		5312	5596	Association	12887920	Affinity Capture-MS	19615732	
028	CASP10_DED1-DED2	039	CFLAR_DED1-DED2	Homotypic		8826	24,620	Association	23541952	Affinity Capture-MS	21303910	23070002
028	CASP10_DED1-DED2	051	FADD_DED	Homotypic		6358	31,336	Direct interaction	11717445	Affinity Capture-MS	19615732	9184224
028	CASP10_DED1-DED2	052	FADD_DED-DD			10,204	20,660	Direct interaction	11717445	Affinity Capture-MS	19615732	9184224
030	CASP2_CARD	041	CRADD_CARD-DD			14,156	2416	Association	11156409	Affinity Capture-MS	20562859	
033	CASP8_DED1	061	IRAK3_DD	Heterotypic		968	1346	N/A		N/A		30372424
034	CASP8_DED2	039	CFLAR_DED1-DED2	Homotypic		9886	5338	Protein cleavage	12887920	Affinity Capture-MS	21303910	
035	CASP8_DED1-DED2	039	CFLAR_DED1-DED2	Homotypic		12,804	4088	Protein cleavage	12887920	Affinity Capture-MS	21303910	
035	CASP8_DED1-DED2	051	FADD_DED	Homotypic		6866	6856	Protein cleavage	12887920	Affinity Capture-MS	19615732	9184224
035	CASP8_DED1-DED2	052	FADD_DED-DD			9378	11,018	Protein cleavage	12887920	Affinity Capture-MS	19615732	9184224
039	CFLAR_DED1-DED2	051	FADD_DED	Homotypic		2848	20,516	Association	17047155	Affinity Capture-MS	19369198	9184224
039	CFLAR_DED1-DED2	052	FADD_DED-DD			14,072	20,116	Association	17047155	Affinity Capture-MS	19369198	9184224
042	CRADD_DD	058	IRAK1_DD	Homotypic		1632	964	N/A		N/A		Direct interaction not approved
043	DAPK1_DD	097	PYDC2_PYD	Heterotypic		3536	970	N/A		N/A		Not found
050	FADD_DD	103	RIPK1_DD	Homotypic		3828	52,618	Association	19524513	Affinity Capture-MS	26186194	9184224
050	FADD_DD	111	TRADD_DD	Homotypic		1384	14,500	Association	30561431	Affinity Capture-MS	21145461	9184224
052	FADD_DED-DD	103	RIPK1_DD			20,016	30,588	Association	19524513	Affinity Capture-MS	26186194	8947041
052	FADD_DED-DD	111	TRADD_DD			39,570	31,748	Association	30561431	Affinity Capture-MS	21145461	8565075
052	FADD_DED-DD	108	TNFRSF1A_DD			964	5568	Physical association	30561431	Affinity Capture-Western	8565075	
058	IRAK1_DD	059	IRAK2_DD	Homotypic		5756	1960	Physical association	10383454	Affinity Capture-MS	26186194	
058	IRAK1_DD	061	IRAK4_DD	Homotypic		1644	1630	Physical association	12860405	Affinity Capture-MS	26496610	
058	IRAK1_DD	067	MYD88_DD	Homotypic		4038	2594	Physical association	17567694	Affinity Capture-MS	22623428	
058	IRAK1_DD	083	NLRP4_PYD	Heterotypic		980	1062	N/A		N/A		Not found
058	IRAK1_DD	087	NLRP8_PYD	Heterotypic		1158	2224	N/A		N/A		Not found
058	IRAK1_DD	110	TNFRSF25_DD	Homotypic		958	976	N/A		N/A		Direct interaction not approved
061	IRAK4_DD	088	NLRP9_PYD	Heterotypic		1176	1072	N/A		N/A		Not found
065	PYRIN_PYD	083	NLRP4_PYD	Homotypic		1280	3860	N/A		N/A		Not found
065	PYRIN_PYD	088	NLRP9_PYD	Homotypic		1064	4656	N/A		N/A		Not found
067	MYD88_DD	071	NLRC3_CARD	Heterotypic		1144	4342	N/A		N/A		Not found
067	MYD88_DD	091	NOD2_CARD2	Heterotypic		1432	3318	N/A		N/A		Direct interaction not approved
071	NLRC3_CARD	083	NLRP4_PYD	Heterotypic		1674	976	N/A		N/A		Not found
073	NLRC5_CARD	088	NLRP9_PYD	Heterotypic		968	1088	N/A		N/A		Not found
080	NLRP14_PYD	083	NLRP4_PYD	Homotypic		1166	998	N/A		N/A		Not found
080	NLRP14_PYD	088	NLRP9_PYD	Homotypic		1044	970	N/A		N/A		Not found
083	NLRP4_PYD	088	NLRP9_PYD	Homotypic		2468	4868	N/A		N/A		Not found
103	RIPK1_DD	108	TNFRSF1A_DD	Homotypic		3886	11,094	Physical association	16611992	Affinity Capture-MS	21145461	9184224
103	RIPK1_DD	110	TNFRSF25_DD	Homotypic		1762	1122	N/A		Affinity Capture-MS	26186194	
103	RIPK1_DD	111	TRADD_DD	Homotypic		6130	3896	Physical association	8612133	Affinity Capture-MS	18655028	9184224
108	TNFRSF1A_DD	110	TNFRSF25_DD	Homotypic		13,248	3080	N/A		N/A		9184224
108	TNFRSF1A_DD	111	TRADD_DD	Homotypic		33,190	43,604	Physical association	7758105	Affinity Capture-MS	21670149	9184224
110	TNFRSF25_DD	111	TRADD_DD	Homotypic		19,914	55,078	N/A		Affinity Capture-Luminescence	22939624	9184224

Combinations with ALPHA signals higher than 1 × 10^3^ RLU in both FLAG × biotin and biotin × FLAG pairs, including 57 domain–domain pairs (53 protein–protein pairs). The interaction information was obtained from the following databases: VaProS (https://vapros.org/) and BioGRID (https://thebiogrid.org/). Representative references are listed.

### Asymmetrically distributed nonself-interacting pairs in the heat map indicate possible interactions that require further confirmation

Some points were asymmetrically distributed across the heat map and there was no corresponding symmetric interaction on the opposite side of the diagonal (Fig. 3, hereafter called one-sided interactions). All such one-sided interactions that had ALPHA signals higher than 2 × 10^3^ RLU with either the FLAG × biotin or biotin × FLAG pair were extracted and listed in Table 2. Among the 129 pairs, 45 were homotypic and 80 were heterotypic. Furthermore, 17 interactions have been previously reported, including 8 homotypic interactions and 4 heterotypic interactions.

**Table 2:** Combinations hinting at possible interactions.

Combination of death domains					AlphaScreen signal (RLU)	Interaction			
Biotin-tagged domain		FLAG-tagged domain		Homo/hetero		VaProS (updated: December 8, 2020)		BioGRID (build 4.1.190)	
No.	Name	No.	Name			Description	PubMed ID	Interaction confirmed by	PubMed ID
003	ANK2_DD	015	CARD10_CARD	Heterotypic	3296	N/A		N/A	
003	ANK2_DD	047	DTHD1_DD	Homotypic	11,380	N/A		N/A	
003	ANK2_DD	111	TRADD_DD	Homotypic	2292	N/A		N/A	
005	ANKDD1A_PYD	042	CRADD_DD	Heterotypic	2906	N/A		N/A	
005	ANKDD1A_PYD	043	DAPK1_DD	Heterotypic	2558	N/A		N/A	
005	ANKDD1A_PYD	058	IRAK1_DD	Heterotypic	5272	N/A		N/A	
005	ANKDD1A_PYD	061	IRAK4_DD	Heterotypic	2228	N/A		N/A	
005	ANKDD1A_PYD	091	NOD2_CARD2	Heterotypic	2254	N/A		N/A	
005	ANKDD1A_PYD	115	UNC5D_DD	Heterotypic	3122	N/A		N/A	
009	ASC_PYD	011	BCL10_CARD	Heterotypic	7302	Association	22267217	N/A	
009	ASC_PYD	065	PYRIN_PYD	Homotypic	4142	Physical association	25006247	N/A	
009	ASC_PYD	076	NLRP10_PYD	Homotypic	5626	N/A		N/A	
009	ASC_PYD	082	NLRP3_PYD	Homotypic	3288	Colocalization	24630722	N/A	
009	ASC_PYD	096	PYDC1_PYD	Homotypic	4236	Colocalization	17178784	N/A	
012	BFAR_DED	042	CRADD_DD	Heterotypic	2784	N/A		N/A	
012	BFAR_DED	058	IRAK1_DD	Heterotypic	3826	N/A		N/A	
012	BFAR_DED	061	IRAK4_DD	Heterotypic	2286	N/A		N/A	
012	BFAR_DED	062	LRRD1_DD	Heterotypic	2362	N/A		N/A	
012	BFAR_DED	071	NLRC3_CARD	Heterotypic	2740	N/A		N/A	
012	BFAR_DED	088	NLRP9_PYD	Heterotypic	2282	N/A		N/A	
012	BFAR_DED	115	UNC5D_DD	Heterotypic	3026	N/A		N/A	
016	CARD11_CARD	015	CARD10_CARD	Homotypic	4008	N/A		N/A	
017	CARD14_CARD	058	IRAK1_DD	Heterotypic	2032	N/A		N/A	
018	CARD16_CARD	020	CARD18_CARD	Homotypic	2064	N/A		N/A	
020	CARD18_CARD	072	NLRC4_CARD	Homotypic	3806	N/A		N/A	
021	CARD19_CARD	011	BCL10_CARD	Homotypic	2964	Colocalization	15637807	N/A	
021	CARD19_CARD	015	CARD10_CARD	Homotypic	4128	N/A		N/A	
021	CARD19_CARD	030	CASP2_CARD	Homotypic	2710	N/A		N/A	
021	CARD19_CARD	052	FADD_DED-DD	Heterotypic	7894	N/A		N/A	
021	CARD19_CARD	064	MAVS_CARD	Homotypic	31,768	N/A		N/A	
021	CARD19_CARD	088	NLRP9_PYD	Heterotypic	2092	N/A		N/A	
021	CARD19_CARD	108	TNFRSF1A_DD	Heterotypic	31,120	N/A		N/A	
023	CARD8_CARD	072	NLRC4_CARD	Homotypic	5474	N/A		N/A	
026	CASP10_DED1	037	CFLAR_DED1	Homotypic	5528	Association	12887920	Affinity Capture-MS	21303910
026	CASP10_DED1	042	CRADD_DD	Heterotypic	3234	N/A		N/A	
026	CASP10_DED1	047	DTHD1_DD	Heterotypic	4662	N/A		N/A	
026	CASP10_DED1	071	NLRC3_CARD	Heterotypic	4320	N/A		N/A	
026	CASP10_DED1	115	UNC5D_DD	Heterotypic	2982	N/A		N/A	
029	CASP12_CARD	011	BCL10_CARD	Homotypic	4998	N/A		N/A	
029	CASP12_CARD	037	CFLAR_DED1	Heterotypic	2042	N/A		N/A	
029	CASP12_CARD	042	CRADD_DD	Heterotypic	3880	N/A		N/A	
029	CASP12_CARD	044	DEDD_PYD	Heterotypic	2244	N/A		N/A	
029	CASP12_CARD	047	DTHD1_DD	Heterotypic	2052	N/A		N/A	
029	CASP12_CARD	061	IRAK4_DD	Heterotypic	2518	N/A		N/A	
029	CASP12_CARD	062	LRRD1_DD	Heterotypic	2330	N/A		N/A	
029	CASP12_CARD	071	NLRC3_CARD	Homotypic	2600	N/A		N/A	
029	CASP12_CARD	088	NLRP9_PYD	Heterotypic	4390	N/A		N/A	
029	CASP12_CARD	115	UNC5D_DD	Heterotypic	2976	N/A		N/A	
033	CASP8_DED1	058	IRAK1_DD	Heterotypic	2774	N/A		N/A	
033	CASP8_DED1	071	NLRC3_CARD	Heterotypic	2390	N/A		N/A	
034	CASP8_DED2	038	CFLAR_DED2	Homotypic	2734	N/A		Affinity Capture-MS	21303910
034	CASP8_DED2	051	FADD_DED	Heterotypic	4662	Association	18946037	Affinity Capture-MS	19615732
034	CASP8_DED2	052	FADD_DED-DD		4062	Association	18946037	Affinity Capture-MS	19615732
034	CASP8_DED2	071	NLRC3_CARD	Heterotypic	2000	N/A		N/A	
036	CASP9_CARD	011	BCL10_CARD	Homotypic	2182	N/A		N/A	
036	CASP9_CARD	049	EDARADD_DD	Heterotypic	2740	N/A		N/A	
036	CASP9_CARD	064	MAVS_CARD	Homotypic	28,754	N/A		N/A	
045	DEDD2_DED	044	DEDD_PYD	Heterotypic	2556	N/A		Affinity Capture-MS	19738201
048	EDAR_DD	011	BCL10_CARD	Heterotypic	2040	N/A		N/A	
050	FADD_DD	108	TNFRSF1A_DD	Homotypic	5854	Physical association	16611992	Affinity Capture-MS	19615732
053	FAS_DD	052	FADD_DED-DD		38,730	Physical association	7536190	Affinity Capture-MS	19615732
053	FAS_DD	103	RIPK1_DD	Homotypic	8838	Physical association	7538908	Affinity Capture-MS	19940151
054	IFI16_PYD	103	RIPK1_DD	Heterotypic	2604	N/A		N/A	
055	IFIH1_CARD1	042	CRADD_DD	Heterotypic	2136	N/A		N/A	
055	IFIH1_CARD1	058	IRAK1_DD	Heterotypic	2690	N/A		N/A	
055	IFIH1_CARD1	071	NLRC3_CARD	Homotypic	3378	N/A		N/A	
055	IFIH1_CARD1	115	UNC5D_DD	Heterotypic	2366	N/A		N/A	
057	IFIH1_CARD1-CARD2	071	NLRC3_CARD	Homotypic	3528	N/A		N/A	
059	IRAK2_DD	042	CRADD_DD	Homotypic	2948	N/A		N/A	
059	IRAK2_DD	047	DTHD1_DD	Homotypic	2184	N/A		N/A	
059	IRAK2_DD	062	LRRD1_DD	Homotypic	2138	N/A		N/A	
059	IRAK2_DD	071	NLRC3_CARD	Heterotypic	3100	N/A		N/A	
059	IRAK2_DD	115	UNC5D_DD	Homotypic	3102	N/A		N/A	
062	LRRD1_DD	011	BCL10_CARD	Heterotypic	2252	N/A		N/A	
065	PYRIN_PYD	009	ASC_PYD	Homotypic	2938	Physical association	11498534	N/A	
065	PYRIN_PYD	010	ASC_PYD-CARD		2908	Physical association	11498534	N/A	
065	PYRIN_PYD	020	CARD18_CARD	Heterotypic	5470	N/A		N/A	
065	PYRIN_PYD	021	CARD19_CARD	Heterotypic	6436	N/A		N/A	
067	MYD88_DD	042	CRADD_DD	Homotypic	3134	N/A		N/A	
067	MYD88_DD	047	DTHD1_DD	Homotypic	2670	N/A		N/A	
067	MYD88_DD	115	UNC5D_DD	Homotypic	3966	N/A		N/A	
072	NLRC4_CARD	011	BCL10_CARD	Homotypic	3282	N/A		Affinity Capture-MS	21907836
081	NLRP2_PYD	106	TNFRSF10B_DD	Heterotypic	7216	N/A		N/A	
083	NLRP4_DD	004	ANK3_DD	Homotypic	2058	N/A		N/A	
083	NLRP4_DD	011	BCL10_CARD	Heterotypic	2844	N/A		N/A	
083	NLRP4_DD	020	CARD18_CARD	Heterotypic	8070	N/A		N/A	
083	NLRP4_DD	021	CARD19_CARD	Heterotypic	5258	N/A		N/A	
083	NLRP4_DD	044	DEDD_PYD	Heterotypic	2948	N/A		N/A	
086	NLRP7_PYD	037	CFLAR_DED1	Heterotypic	6814	N/A		N/A	
087	NLRP8_PYD	071	NLRC3_CARD	Heterotypic	2348	N/A		N/A	
087	NLRP8_PYD	115	UNC5D_DD	Heterotypic	2460	N/A		N/A	
088	NLRP9_PYD	011	BCL10_CARD	Heterotypic	2936	N/A		N/A	
088	NLRP9_PYD	020	CARD18_CARD	Heterotypic	5886	N/A		N/A	
088	NLRP9_PYD	021	CARD19_CARD	Heterotypic	4226	N/A		N/A	
088	NLRP9_PYD	084	NLRP5_PYD	Homotypic	2852	N/A		N/A	
089	NOD1_CARD	071	NLRC3_CARD	Homotypic	2086	N/A		N/A	
090	NOD2_CARD1	015	CARD10_CARD	Homotypic	2438	N/A		N/A	
090	NOD2_CARD1	042	CRADD_DD	Heterotypic	2738	N/A		N/A	
090	NOD2_CARD1	071	NLRC3_CARD	Homotypic	3656	N/A		N/A	
090	NOD2_CARD1	115	UNC5D_DD	Heterotypic	2200	N/A		N/A	
092	NOD2_CARD1-CARD2	015	CARD10_CARD	Homotypic	10,988	N/A		N/A	
092	NOD2_CARD1-CARD2	037	CFLAR_DED1	Heterotypic	4002	N/A		N/A	
092	NOD2_CARD1-CARD2	042	CRADD_DD	Heterotypic	2170	N/A		N/A	
092	NOD2_CARD1-CARD2	071	NLRC3_CARD	Homotypic	8834	N/A		N/A	
092	NOD2_CARD1-CARD2	073	NLRC5_CARD	Homotypic	3466	N/A		N/A	
095	PIDD1_DD	015	CARD10_CARD	Heterotypic	5146	N/A		N/A	
095	PIDD1_DD	111	TRADD_DD	Homotypic	4018	N/A		N/A	
097	PYDC2_PYD	011	BCL10_CARD	Heterotypic	5342	N/A		N/A	
097	PYDC2_PYD	037	CFLAR_DED1	Heterotypic	9994	N/A		N/A	
097	PYDC2_PYD	042	CRADD_DD	Heterotypic	4782	N/A		N/A	
097	PYDC2_PYD	047	DTHD1_DD	Heterotypic	4984	N/A		N/A	
097	PYDC2_PYD	058	IRAK1_DD	Heterotypic	7260	N/A		N/A	
097	PYDC2_PYD	062	LRRD1_DD	Heterotypic	2632	N/A		N/A	
097	PYDC2_PYD	071	NLRC3_CARD	Heterotypic	8938	N/A		N/A	
097	PYDC2_PYD	091	NOD2_CARD2	Heterotypic	3810	N/A		N/A	
097	PYDC2_PYD	105	TNFRSF10A_DD	Heterotypic	3174	N/A		N/A	
097	PYDC2_PYD	115	UNC5D_DD	Heterotypic	4210	N/A		N/A	
100	RIG-I_CARD1	042	CRADD_DD	Heterotypic	2438	N/A		N/A	
100	RIG-I_CARD1	071	NLRC3_CARD	Homotypic	2304	N/A		N/A	
100	RIG-I_CARD1	115	UNC5D_DD	Heterotypic	2032	N/A		N/A	
101	RIG-I_CARD2	042	CRADD_DD	Heterotypic	2016	N/A		N/A	
101	RIG-I_CARD2	058	IRAK1_DD	Heterotypic	3274	N/A		N/A	
101	RIG-I_CARD2	071	NLRC3_CARD	Homotypic	3648	N/A		N/A	
101	RIG-I_CARD2	091	NOD2_CARD2	Homotypic	2020	N/A		N/A	
101	RIG-I_CARD2	115	UNC5D_DD	Heterotypic	3188	N/A		N/A	
102	RIG-I_CARD1-CARD2	037	CFLAR_DED1	Heterotypic	2738	N/A		N/A	
107	TNFRSF11B_CARD	103	RIPK1_DD	Heterotypic	2722	N/A		N/A	
108	TNFRSF1A_DD	011	BCL10_CARD	Heterotypic	4508	N/A		Affinity Capture-MS	21903422
110	TNFRSF25_DD	052	FADD_DED-DD		3382	N/A		N/A	

Combinations were extracted where AlphaScreen signals of more than 2 × 103 RLU were observed in either FLAG × biotin or biotin × FLAG pair.

### The strength of ALPHA signals does not correlate with the expression levels of domains, but with specific pair combinations

**Fig. 6:**
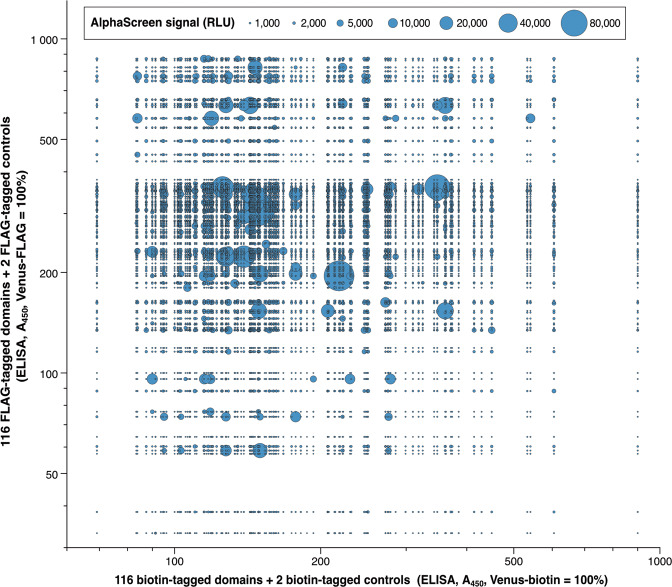
The strength of ALPHA signals is not correlated with the expression levels of the domains, but with specific pair combinations. A bubble chart compares the domain expression levels determined by ELISAs and the strength of ALPHA signals. Blue bubbles show ALPHA signals. The bigger the bubble area, the stronger the signal. The longitudinal axis shows relative concentrations of FLAG-tagged domains and the horizontal axis shows relative concentrations of biotin-tagged domains.

To investigate whether the non-normalized expression levels of DDSP domains affect the ALPHA results in an unbiased fashion, we compared the expression levels of the domains and the distribution of positive ALPHA signals. In the bubble chart in Fig. 6, blue bubbles show ALPHA signals. The bigger the bubble area, the stronger the signal. The longitudinal axis shows relative concentrations of FLAG-tagged domains and the horizontal axis shows relative concentrations of biotin-tagged domains. Significantly large bubbles were widely and randomly scattered across the chart, instead of gathering in specific regions, such as the top right where the expressed domains were most abundant. In addition, the sizes of the bubbles showed no linear correlation with the expression levels of domains. These results demonstrate that the strength of ALPHA signals was not correlated with the expression levels of the domains, but with specific pair combinations.

### Co-immunoprecipitation confirms previously reported interactions and provides hints about new interactions

The ALPHA is an excellent technology to analyze protein–protein interactions because it is homogeneous, highly sensitive, and convenient. However, doubts remain about whether and to what extent the ALPHA results reflect and are consistent with the real situations in natural cells. To validate the accuracy and veracity of the ALPHA results, we performed a co-immunoprecipitation assay.

**Fig. 7:**
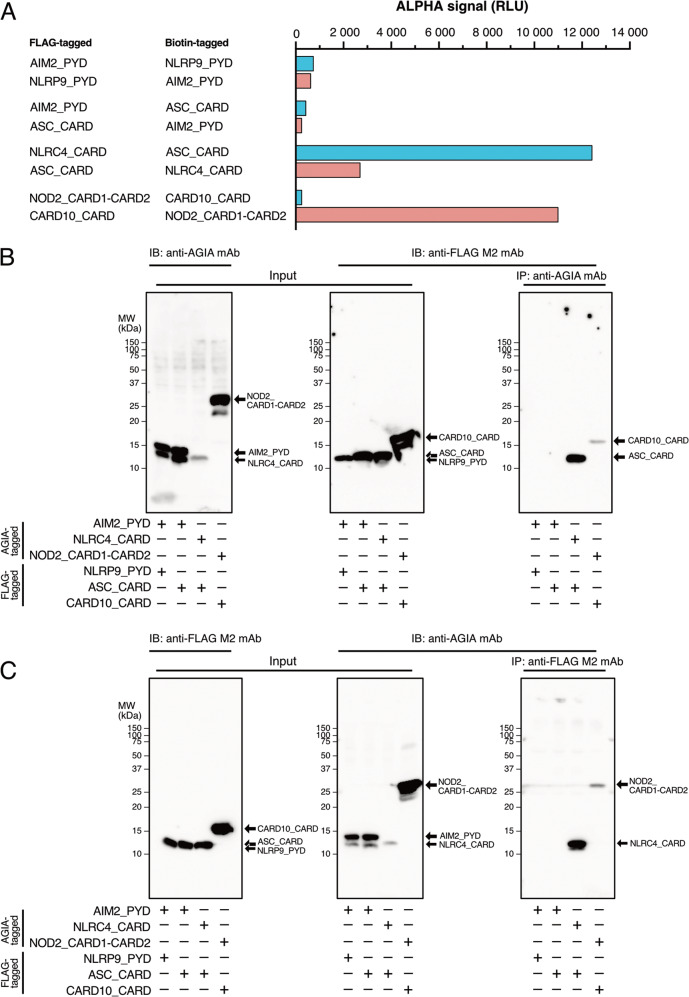
An immunoprecipitation assay confirms the ALPHA results. **A** ALPHA results of the four domain–domain interaction pairs examined in panels **B** and **C**. **B**, **C** An AGIA-tagged domain and a FLAG-tagged domain were co-expressed in HEK293T cells. Soluble supernatants generated from whole cell lysates (input) were applied to immunoprecipitation assay using an anti-AGIA or an anti-FLAG antibody, and co-immunoprecipitants were detected by immunoblotting with an anti-FLAG or anti-AGIA antibody, respectively.

We selected eight representative pairs of domains from the 116 domains according to the ALPHA (Fig. 7A). Among them, four pairs between AIM2_PYD × NLRP9_PYD and AIM2_PYD × ASC_CARD had low signals, indicating these domains do not interact [13]. The two pairs between (NLRC4_CARD × ASC_CARD) both demonstrated significantly high signals, indicating that these domains interact, which actually was reported as components of NLRC4 inflammasome (Table 1) [21]. A one-sided pair (CARD10_CARD-FLAG × NOD2_CARD1-CARD2-biotin) exhibited an extremely high signal, whereas the other pair with reversed tags did not. An interaction between CARD10_CARD and NOD2_CARD1-CARD2 has not been previously reported and therefore needed to be confirmed in cells.

The results of the co-immunoprecipitation assay are shown in Fig. 7B, C. As expected, a homotypic domain pair (AIM2_PYD × NLRP9_PYD) that had low ALPHA signals and a known noninteracting heterotopic domain pair (AIM2_PYD × ASC_CARD) did not co-immunoprecipitate [22], whereas a known interacting homotypic domain pair (NLRC4_CARD × ASC_CARD) did [23]. These immunoprecipitation results were consistent with those of the ALPHA assay and previous reports. The homotypic domain pair of CARD10_CARD × NOD2_CARD1-CARD2, which had a high signal in the ALPHA assay, were reciprocally co-immunoprecipitated (Fig. 7B, C).

## Discussion

Human DDSPs are involved in assembly of multimeric complexes associated with signaling cascades that lead to cell death and inflammation [3]. Disruption of this assembly or dysregulation of DDSP interactions due to inherited gene mutations causes immunodeficiency and/or autoinflammatory diseases [4]. However, the relationships between the genotypes and phenotypes of inherited immunodeficiency and/or autoinflammatory diseases are not fully understood. In some cases, even though the responsible genes have been reported, mutations of these genes have not been found [12]. Thus, we hypothesize that DDSP interactions are involved in some unknown signaling cascades. In this study, we comprehensively analyzed the interactions of DDSP domains to explore novel interaction pathways.

The greatest difficulties faced when comprehensively investigating the interactions of DDSP domains are the preparation of more than 100 different kinds of proteins and the conduction of a one-against-all domain–domain interaction assay, where conventional methods are commonly used such as yeast two-hybrid assay, pull-down assay, and immunoprecipitation assay. Here, we used the wheat germ cell-free protein synthesis system and ALPHA, which greatly facilitated our study. We successfully synthesized 116 FLAG-tagged and 116 biotin-tagged domains of DDSPs using the wheat germ cell-free protein synthesis system (Supplementary Table S1) and comprehensively analyzed domain–domain interactions using ALPHA (Fig. 3). The ALPHA results of domain–domain interactions were reliable and trustworthy. An MA plot showed high reproducibility (98.74%) between two repeats (Fig. 4). A bubble chart demonstrated that the expression levels of DDSP domains did not affect the strength of ALPHA signals (Fig. 6). Furthermore, many domain interactions detected in this study were previously reported (Tables 1 and  2).

Our data showed that 32.8% of self-interacting pairs and 5.7% of nonself-interacting pairs yielded positive results in the ALPHA, indicating that self-interacting pairs interact more readily than nonself-interacting pairs. Although two domains contained heterogeneous DFDs, all self-interacting pairs exhibited homotypic binding [24].

DDSP domains are widely believed to exert their effects via monovalent, homotypic, subfamily-restricted interactions (DD × DD, CARD × CARD, DED × DED, and PYD × PYD), generating large multi-subunit structures comprising only one type of protein [25]. However, heterotypic binding has been reported for some exceptional DDSP domains [26]. We found ten domain pairs as novel candidates for heterotypic interactions (Fig. 5 and Table 1). Such interactions may help to elucidate the molecular basis of signaling complexes and pathways that regulate cell death and inflammation.

This study shows many novel possible interactions (Tables 1 and 2). Twenty novel double-sided interactions (Fig. 5) were considered reliable because many of the other double-sided interactions identified in this study were previously reported. There were several combinations for which no direct interaction has been previously demonstrated, including CARD16_CARD × NLRC4_CARD, CRADD_DD × IRAK1_DD, IRAK1_DD × TNFRSF25_DD, and MYD88_DD × NOD2_CARD2 (Table 1), even though previous reports suggested their biological and pathological relevance. For example, acute coronary syndrome (ACS) is a disorder in which blood supply to the heart is suddenly blocked, leading to heart attacks and unstable angina. A genome-wide association study of 18,624 patients with ACS identified the associated gene loci in IL-18, NLRC4, and CARD16; however, direct interactions between these proteins have not been demonstrated [27]. Our study identified a double-sided interaction between CARD16_CARD and NLRC4_CARD (Fig. 5 and Table 1). The direct interaction between NLRC4 and CARD16 highlights the role of NLRC4 inflammasome regulation in ACS. Moreover, many studies suggest that Toll-like receptors (TLRs) and nucleotide-binding oligomerization domain 1 (NOD1) and NOD2 synergize with each other to induce production of cytokines and antimicrobial peptides. However, the molecular mechanisms underlying this synergy have not been elucidated [28]. The synergic effect of TLRs and NODs leads to poor outcomes in individuals with septic shock syndrome caused by Gram-positive or -negative bacterial infections [29, 30]. The double-sided interaction between MYD88_DD and NOD2_CARD2 (the second CARD domain from the N-terminus) identified in this study may support the hypothesis that there are direct crosstalk between signaling pathways downstream of NODs and TLRs (Table 1). In addition, our study indicated the direct interaction between IRAK1 and CRADD (Fig. 5 and Table 1), supporting the previous report that IRAK1 functions to inhibit radiation therapy-induced apoptosis mediated by the PIDDosome (PIDD-RAIDD-caspase-2) [31].

The one-sided interactions listed in Table 2, few of which were previously reported, are supposedly less reliable than the double-sided interactions listed in Table 1. The interactions need to be confirmed by co-immunoprecipitation assays in human cell lines. Many immunoprecipitation experiments assessing DDSP interactions have been performed using HEK293T cells. Therefore, we used HEK293T cells and compared the novel interaction between NOD2_CARD1-CARD2 and CARD10_CARD and the known interaction between NLRC4_CARD and ASC_CARD. NOD2_CARD1-CARD2-AGIA was co-immunoprecipitated by CARD10_CARD-FLAG and vice versa when the proteins were co-expressed, which was consistent with the ALPHA results (Fig. 7B, C and Supplementary Table S3), suggesting that CARD10 interacts with NOD2. Consequently, it is worth testing the other one-sided interactions listed in Table 2.

CARD10 (also known as CARD and membrane-associated guanylate kinase (MAGUK) domain-containing protein 3 (CARMA3)) functions as a scaffold and is involved in NF-κB activation in response to various types of upstream innate immune signaling or modulates the interactions of deubiquitinating enzymes such as A20 and CYLD [32]. Several amino acid mutations of CARD10 are reportedly responsible for some inflammatory bowel diseases [33]. NOD2 is a Nod-like receptor that recognizes the bacterial peptidoglycan component muramyl dipeptide, leading to NF-κB activation [34, 35]. Gain-of-function mutations of NOD2 lead to autoinflammatory diseases such as early-onset sarcoidosis and Blau syndrome [36]. Loss-of-function mutations of NOD2 lead to susceptibility to an inflammatory bowel disease called Crohn’s disease [37, 38].

Although the results of this study were highly reproducible, there are several limitations. First, all the recombinant DDSP domains synthesized and used in this study were fused with tags. The type and position of the tag may affect the ALPHA results. Depending on the structure of the domain, the fused tag may be concealed inside the mature domain and inaccessible to the detection antibody or streptavidin. Furthermore, when a domain interacts with partners via amino acid residues close to the terminus, the tag may interfere with the interaction, leading to false ALPHA results. This may explain one of the cause of known pairs of interactors that do not have a high enough ALPHA signal (Supplementary Table S4). Therefore, it is recommended that domains with tags fused at different positions are examined before conducting the ALPHA. The domain interactions were not noticeably affected according to whether the tag was located at the N- or C-terminus. Therefore, we tagged the C-termini of all domains with FLAG or biotin to ensure the domains and tags were fully translated. Second, the recombinant DDSP domains were synthesized using the wheat cell-free synthesis system and therefore their structures may differ from those of proteins expressed in cells. In particular, proteins synthesized using cell-free systems possibly have errors in post-translational modifications. For DDSP domains that require a certain post-translational modification for signal transduction, it is recommended that enzymes that catalyze such modifications, such as protein kinases, are added to the cell-free system. Finally, although we analyzed interactions for all possible DDSP domains combinations, some interactions may not occur in cells. Two DDSPs with different subcellular localizations and expression profiles are very unlikely to encounter each other in cells. Regarding the new interacting pairs identified by our comprehensive analysis, their localizations and interactions should be confirmed and overlap of their temporal and spatial expression patterns should be verified. Therefore, the ALPHA results could not be quantitatively analyzed to compare the strengths of the domain–domain interactions.

In conclusion, we believe that our comprehensive investigation of DDSPs will be helpful for the field of DDSP-related diseases, especially immunodeficiency and autoinflammatory diseases. This work may facilitate future research aiming to identify pharmaceutical targets for drug discovery and to elucidate the pathogenesis of these diseases.

## Supplementary information

Supplementary Figure S1

Supplementary Table S1

Supplementary Table S2

Supplementary Table S3

Supplementary Table S4
