# Supplementary figures and images for: A comprehensive interaction study provides a potential domain interaction network of human death domain superfamily proteins
Source: Cell Death Differ. 2021 May 15;28(11):2991–3008. doi: 10.1038/s41418-021-00796-x (PMC8564539; doi:10.1038/s41418-021-00796-x)

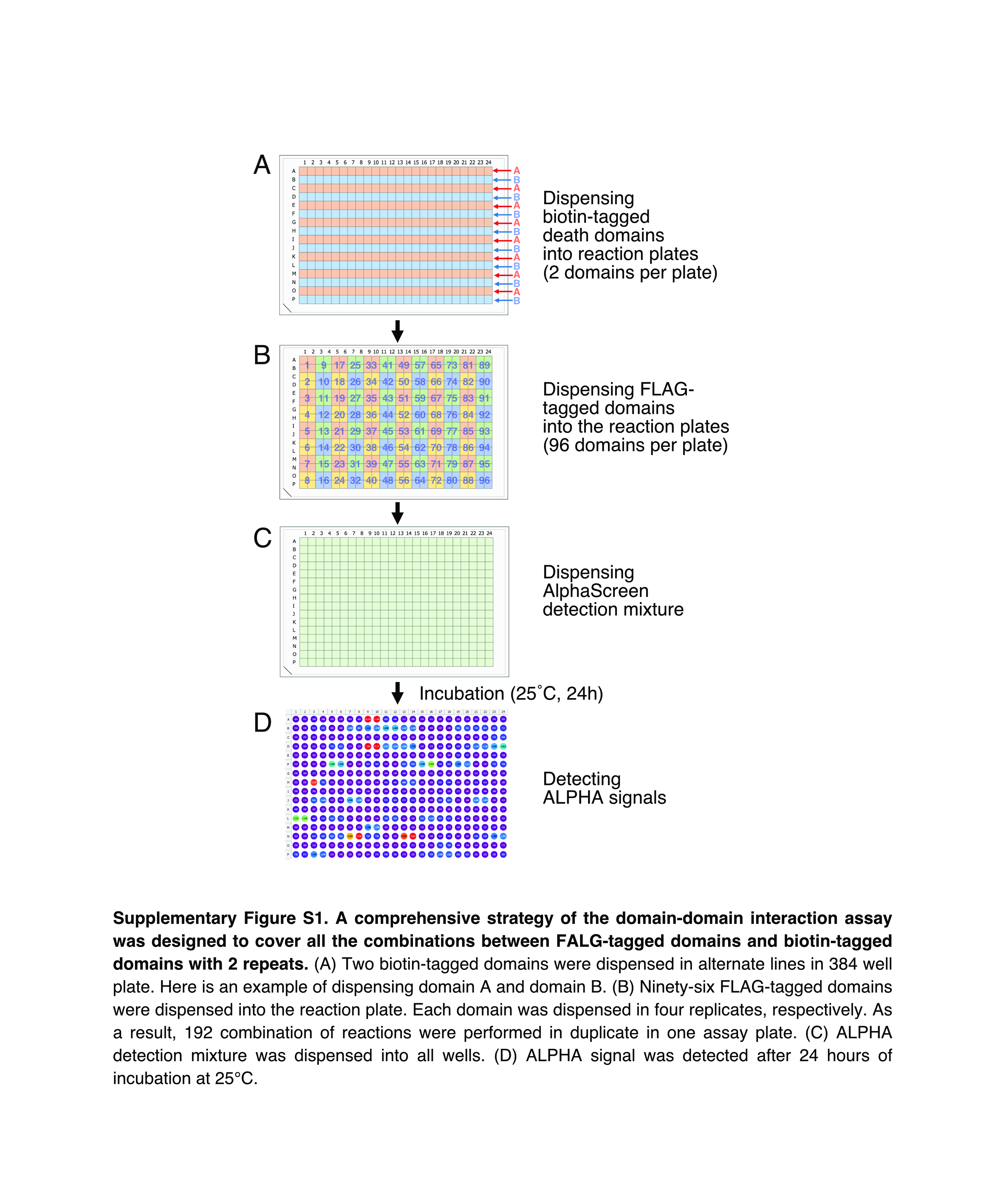

Supplement: Supplementary file 1 — Supplementary Figure S1 [file 41418_2021_796_MOESM1_ESM.tif]
